# Targeting of the Lipid Metabolism Impairs Resistance to BRAF Kinase Inhibitor in Melanoma

**DOI:** 10.3389/fcell.2022.927118

**Published:** 2022-07-13

**Authors:** Elisabetta Vergani, Giovanni L. Beretta, Mariachiara Aloisi, Matteo Costantino, Cristina Corno, Simona Frigerio, Stella Tinelli, Matteo Dugo, Felice Maria Accattatis, Agnese Granata, Lorenzo Arnaboldi, Monica Rodolfo, Paola Perego, Laura Gatti

**Affiliations:** ^1^ Unit of Immunotherapy of Human Tumors, Department of Research, Fondazione IRCCS Istituto Nazionale dei Tumori di Milano, Milan, Italy; ^2^ Unit of Molecular Pharmacology, Department of Applied Research and Technological Development, Fondazione IRCCS Istituto Nazionale dei Tumori, Milan, Italy; ^3^ Department of Medical Oncology, IRCCS Ospedale San Raffaele, Milan, Italy; ^4^ Department of Pharmacy, Health and Nutritional Sciences, University of Calabria, Cosenza, Italy; ^5^ Department of Pharmacological and Biomolecular Sciences DISFeB, Università degli Studi di Milano, Milan, Italy; ^6^ Neurobiology Laboratory, Department of Clinical Neurosciences, Fondazione IRCSS Istituto Neurologico Carlo Besta, Milan, Italy

**Keywords:** melanoma, BRAF inhibitors, drug resistance, lipid metabolism, avasimibe

## Abstract

Drug resistance limits the achievement of persistent cures for the treatment of melanoma, in spite of the efficacy of the available drugs. The aim of the present study was to explore the involvement of lipid metabolism in melanoma resistance and assess the effects of its targeting in cellular models of melanoma with acquired resistance to the BRAF-inhibitor PLX4032/Vemurafenib. Since transcriptional profiles pointed to decreased cholesterol and fatty acids synthesis in resistant cells as compared to their parental counterparts, we examined lipid composition profiles of resistant cells, studied cell growth dependence on extracellular lipids, assessed the modulation of enzymes controlling the main nodes in lipid biosynthesis, and evaluated the effects of targeting Acetyl-CoA Acetyltransferase 2 (ACAT2), the first enzyme in the cholesterol synthesis pathway, and Acyl-CoA Cholesterol Acyl Transferase (ACAT/SOAT), which catalyzes the intracellular esterification of cholesterol and the formation of cholesteryl esters. We found a different lipid composition in the resistant cells, which displayed reduced saturated fatty acids (SFA), increased monounsaturated (MUFA) and polyunsaturated (PUFA), and reduced cholesteryl esters (CE) and triglycerides (TG), along with modulated expression of enzymes regulating biosynthetic nodes of the lipid metabolism. The effect of tackling lipid metabolism pathways in resistant cells was evidenced by lipid starvation, which reduced cell growth, increased sensitivity to the BRAF-inhibitor PLX4032, and induced the expression of enzymes involved in fatty acid and cholesterol metabolism. Molecular targeting of ACAT2 or pharmacological inhibition of SOAT by avasimibe showed antiproliferative effects in melanoma cell lines and a synergistic drug interaction with PLX4032, an effect associated to increased ferroptosis. Overall, our findings reveal that lipid metabolism affects melanoma sensitivity to BRAF inhibitors and that extracellular lipid availability may influence tumor cell response to treatment, a relevant finding in the frame of personalized therapy. In addition, our results indicate new candidate targets for drug combination treatments.

## Introduction

Melanoma is a highly aggressive disease accounting for the majority of death for skin cancers. Declines in mortality have accelerated in recent years, likely due to the amelioration of medical management of the disease (Siegel et al., 2021). The activation of the RAS-RAF-MEK-ERK axis is a common event in the disease due to the frequent activating mutation of the BRAF gene. BRAF^V600E^ mutation is carried by the 40% of melanoma and results in constitutive activation of cell survival pathways, a feature mainly responsible for melanoma aggressiveness. The BRAF^V600E^ kinase inhibitor PLX4032 (vemurafenib) demonstrated efficacy in metastatic melanoma patients with improved access to innovative medicines (McArthur et al., 2014; Kandolf Sekulovic et al., 2018). However, treated patients develop resistance to BRAF inhibitors (BRAFi) after 6–9 months (Gershenwald and Scolyer, 2018), through mechanisms reactivating the MAPK pathway. BRAF/MEK co-targeting by the combination of BRAFi with MEK-inhibitors showed increased efficacy, although the development of resistance limits the achievement of persistent cures (Kakadia et al., 2018).

Melanoma driver genes, including BRAF, have been implicated in the regulation of cellular energy metabolism (Abildgaard and Guldberg, 2015), which has been shown to undergo reprogramming, being characterized by plasticity that facilitates melanoma progression and drug resistance (Alkaraki et al., 2021). System biology and experimental approaches support a major role for lipids in cellular homeostasis, with lipid dysfunction occurring during tumor development (Beloribi-Djefaflia et al., 2016; Kopecka et al., 2020). Indeed, lipids take part in oncogenic signaling and may act as inducers of multidrug resistance (Beloribi-Djefaflia et al., 2016; Kopecka et al., 2020). In addition, different fatty acids (FA) participate in modulating tumor cell proliferation (Dierge and Feron, 2019), with an impact of their exogenous levels on cell death pathways (Magtanong et al., 2019). Thus, a better understanding of the molecular bases of drug resistance with specific reference to lipid metabolism may provide new knowledge exploitable for clinical application, as we previously proposed for Fatty Acid Synthase (FASN) and 24-Dehydrocholesterol Reductase (DHCR24) (Stamatakos et al., 2021).

Based on this background, the aim of the present study was to examine lipid metabolism in matched pairs of sensitive and PLX4032-resistant melanoma cell lines, with particular reference to gene expression profiles and lipid composition impact on sensitivity to BRAFi. By this approach, we found that ACAT2, the first enzyme in the cholesterol synthesis pathway, and SOAT, the enzyme acting in the intracellular esterification of cholesterol and the formation of cholesteryl esters, play a role in drug resistance and can be proposed as targets for modulation of BRAFi response in melanoma.

## Materials and Methods

### Cell Lines, Drugs and Cell Proliferation Assays

The melanoma cell lines LM16, LM36, LM47, and the corresponding PLX4032-resistant sublines LM16R, LM36R and LM47R generated upon chronic exposure of parental cells to PLX4032, were derived at the Fondazione IRCCS, Istituto Nazionale dei Tumori of Milan (Daniotti et al., 2004; Vergani et al., 2011; Vergani et al., 2022). All cell lines were cultured in RPMI-1640 medium (Lonza, Basel, Switzerland), supplemented with 10% FBS (Euroclone, Milan, Italy), used within 20 passages from thawing from a frozen stock, tested to verify the maintenance of resistance, routinely checked for mycoplasma contamination (Mycoalert, Lonza), and authenticated using the Stem Elite ID System (Promega, Wisconsin, United States). Lipid starvation was carried out replacing FBS with lipid-depleted serum (S181L, Biowest, Voden Medical Instruments, Meda, Italy). The effect of lipid starvation on cell growth was evaluated by cell sensitivity assays based on cell counting (Z2 Particle Counter, Beckman Coulter, Milan, Italy) over time, after seeding the cells in 6-well plates (2.5 × 10^3^ cells/cm^2^), and by using the CCK8 assay (96992, Sigma-Aldrich, Milan, Italy) which allows to measure cell viability. Exponentially growing cells were seeded into 96-well plates in standard or lipid-free medium and, 24 h later, they were exposed to increasing concentrations of drugs for 48 h. To test the sensitivity to drugs, with and without lipids in culture medium, the number of cells was increased to 12 × 10^3^ cells/well since they did not grow in lipid-free medium at low density. The activation of caspase 3/7 was determined using luminescent Caspase Glo 3/7 assay (G8091, Promega). Cells were seeded (12 × 10^3^ cells/well, 100 µL/well) in 96-well plates and treated with drugs for 48 h in the presence or absence of lipids in the culture medium. Caspase activation was detected according to manufacturer’s instructions.

IC_50_ is defined as the concentration of a drug inhibiting 50% of cell growth/viability. PLX4032 (Selleckchem, Houston, TX, United States) and avasimibe (MedChemExpress, Princeton, NJ, United States) were dissolved and diluted in dimethylsulfoxide (DMSO). Final DMSO concentration in medium never exceeded 0.25%. Data of drug combination assays were analysed according to the Chou–Talalay method (Chou, 2010). The combination index (CI) values were defined using Calcusyn software (Biosoft, Cambridge, UK). CI values lower than 0.85–0.90 indicate synergistic drug interactions, whereas CI values higher than 1.20–1.45 or around 1 define antagonism or additive effect, respectively.

### Ferroptosis Assessment

Cells were seeded in white 96-well plates (12 × 10^3^ cells/well, 100 μL/well) and 24 h later treated with drugs in standard conditions or in lipid-free medium for 24 h. After treatment, the medium was removed and reduced glutathione (GSH) levels were quantified using the GSH/GSSG Glo assay kit, according to the manufacturer’s protocol (V6611, Promega). Luminescence was measured using a plate reader (Tecan, Mannedörf, Switzerland) and concentrations of GSH were calculated through standards provided in the kit. The GSH/GSSG ratio was calculated according to the manufacturer’s guidelines.

### Loss of Function Studies

Cells were plated in 75 cm^2^ flasks (10^4^ cells/cm^2^) and 24 h later transfected using Opti-MEM transfection medium (Thermo Fisher Scientific, Waltham, MA, United States) and RNAiMax (Thermo Fisher Scientific) with 50 nM of two small interfering RNAs (siRNAs) to ACAT2 (Silencer Select siRNA 16900 and 111,620, Thermo Fisher Scientific) or with the mix of the two siRNAs 25 nM each. Control siRNA (Silencer Select Negative Control #2 siRNA, Thermo Fisher Scientific), 50 nM, was used as negative control. The transfection mix was added to cells for 5 h and then it was replaced with standard cell medium. Knockdown efficiency was evaluated by Western blotting 72 h after transfection start. For cell sensitivity assay, transfected cells prepared as above, were re-seeded in 96-well plates at a density of 8 × 10^3^ cells/well in 100 μl and treated with different concentrations of drugs.

### Western Blot Analysis

Western blot analysis was carried out as previously described (Corno et al., 2017). Lysates were fractionated by SDS-PAGE and proteins were blotted on nitrocellulose membranes. Blots were pre-blocked in PBS containing 5% (w/v) dried no fat milk and then incubated overnight at 4°C with antibodies to FASN (HPA006461, Sigma-Aldrich, St. Louis, MO, United States), DHCR24 (2033, Cell Signaling Technology, Danvers, MA, United States), stearoyl–CoA desaturase 1 (SCD1) (ab19862 Abcam, Cambridge, UK), mevalonate kinase (MVK) (ab154515, Abcam), acetyl-CoA acetyltransferase 1 (ACAT1) (ab168342, Abcam), ACAT2 (ab131215, Abcam), glutathione peroxidase 4 (GPX4) (sc166120, Santa Cruz Biotechnology, Dallas, Texas, United States), β-Hydroxy β-methylglutaryl-CoA reductase (HMGCR) (ab174830, Abcam). An anti-β-tubulin antibody (ab6046, Abcam) was used as control for loading. Antibody binding to blots was detected by chemo-luminescence (Amersham Biosciences, Cologno Monzese, Italy). Secondary antibodies were obtained from GE Healthcare.

### Measure of Malondialdehyde Levels

The concentration of malondialdehyde (MDA) was calculated using an ELISA kit from Elabscience (E-EL-0060, Houston, Texas, United States), according to the manufacturer’s protocol. Briefly, sensitive LM16 and LM36 cells, and the corresponding resistant variants were seeded (1 × 10^6^ cells) in 75 cm^2^ flasks in standard or in lipid-free medium and exposed for 24 h to PLX4032 at concentrations corresponding to IC_80_ at 72 h i.e., 0.3 and 23 µM for LM16 and LM16R, respectively, and 0.89 and 13.9 µM for LM36 and LM36R, respectively. Cells were then collected and analyzed according to protocol instructions.

### Lipid Extraction and Analysis

Total lipids were obtained from confluent cells grown 24 h in the absence of serum in 100 mm petri dishes by three consecutive extractions in a cold room with a solution of hexane-isopropanol (3:2) plus butylated hydroxytoluene as antioxidant. In order to quantify the real mass of free and esterified cholesterol and triglycerides, proper amounts of internal standards (stigmasterol, cholesteryl heptadeacanoate and triheptadecanoin) were added to the extraction mixture. While the total FA profile analysis was directly performed on aliquots of the lipid extract, lipid subclasses were separated by seeding aliquots of the extract onto a thin layer chromatography silica gel 60 plate (20 cm × 20 cm with concentrating zone 2.5 cm, Merck, Darmstadt, Germany). After development (eluent hexane/diethylether/acetic acid 80:20:1) and dichlorofluorescein staining, lipid subclasses were identified by comparison of their spots with those of proper standards. Aliquots of total lipid extracts, or portions of silica gel containing the lipids of interest, were collected in sealed glass vials and derivatized by methanolic HCl 3N for 120 min at 80°C or less, as necessary. The obtained methylated FA were extracted by hexane/water and detected by gas-liquid chromatography (GLC) (DANI 1000, DANI, Milan, Italy) equipped with a flame-ionization detector, hydrogen as gas carrier, and a HTA autosampler (HTA instruments, Brescia, Italy). We utilized a MEGA-5 capillary column (0.15 μM film thickness, 0.25 mm internal diameter, 30 m length, MEGA Columns, Legnano, Italy). The oven temperature was programmed as follows: from 150°C to 190°C at 8°C/min; from 190°C to 210°C at 4°C/min, and finally up to 320°C at 12°C/min, hold for 6 min, for a total run of 25.5 min. The identification of each FA was achieved by comparing the retention times of each peak with those of a standardized mixture of methylated fatty acids (FAME MIX 37, Merck). For qualitative analysis, the relative amount of each FA was calculated by the Clarity Software (Clarity, Prague, Czech Republic), by summing the areas under the curve (AUC) of each peak and calculating the relative percentage. The relative percentages in saturated, monounsaturated and polyunsaturated FA were calculated by their sum as 100%. The mass of each FA in CE, TG and that of each lipid subclass were calculated by comparison with the AUC of the internal standard, and normalized by the total cell protein content determined by the bicinchoninic assay. Results are expressed as μg/mg proteins.

### Quantitative Real-Time PCR

Gene expression was determined by qRT-PCR using TaqMan assays. RNA was extracted with miRNeasy Mini kit (Qiagen, Hilden, Germany) and retrotranscribed with the High-Capacity cDNA Archive kit (Thermo Fisher Scientific, Monza, Italy). qRT-PCR was carried out in triplicate and run on the QuantStudio 7 Flex instruments (Thermo Fisher Scientific), and analysis was performed using QuantStudio 6 and 7 Flex software. The results are calculated with the 2-ΔCt method using β-actin or GAPDH as housekeeping gene. Assays for FASN (222990460), SREBF1 (222990448), SREBF2 (222990436) were from Integrated DNA Technology (Coralville, Iowa, United States), and TaqMan assays for DHCR24 (Hs00234140_m1), SCD (Hs01682761_m1), ACAT2 (Hs00255067_m1), MVK (Hs00176077), GAPDH (Hs99999905_m1) and β-actin (4326315 E) were used.

### Gene Expression Analysis

The dataset deposited in the GEO repository (GEO accession number GSE201913) including the transcriptional profiles of six melanoma cell lines and of the relative variants resistant to PLX4032 (LM16/LM16R, LM36/LM36R, LM47/LM47R, LM43/LM43R, LM17/LM17R, LM11/LM11R), was analyzed by Gene Set Enrichment Analysis (GSEA) (Subramanian et al., 2005) and by Metabolizer software (https://metabolizer.babelomics.org), to define enriched Hallmark gene sets and to identify the metabolic modules in gene expression profiles of resistant cell lines (Cubuk et al., 2019).

### Statistical Analysis

All experiments were repeated at least three times. Statistical analysis was performed using GraphPad Prism 5 and 8 (GraphPad Software, United States). The unpaired Student’s t test, one-way and two-way analysis of variance (ANOVA) followed by Bonferroni correction were employed for evaluating significance in difference of means between groups, and values of *p* < 0.05 were considered significant.

## Results

### Dysregulation of Lipid Metabolism Pathways in BRAFi-Resistant Melanoma Cells

**FIGURE 1:**
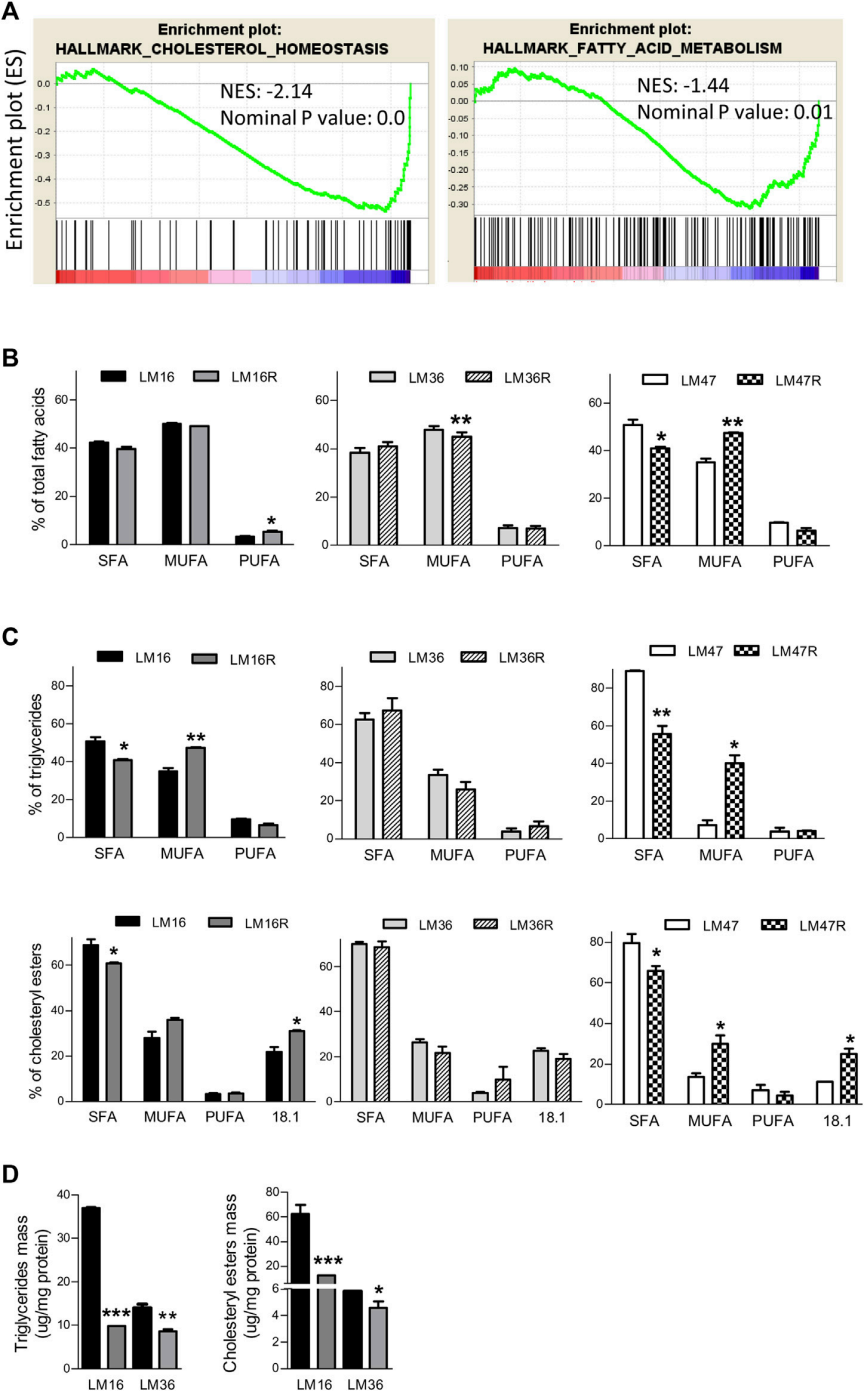
Lipid metabolism alterations in BRAFi-resistant melanoma cell lines. **(A)**. GSEA enrichment plots of lipid metabolism-related gene sets that are downregulated in the BRAFi-resistant cells when compared to the parental cell lines. NES and nominal *p* value are shown. **(B)**. Graph reports relative percentage of SFA, MUFA and PUFA in total FA detected by GLC in LM16/LM16R, LM47/LM47R and LM36/LM36R cell line pairs. **(C)**. percentages of SFA, MUFA and PUFA content in TG and CE. **(D)**. Quantification of TG and CE mass. *p < 0.05, **p < 0.01 and ***p < 0.0001 by unpaired Student’s t test.

To investigate the role of lipid metabolism genes in melanoma resistance to the BRAFi PLX4032, we carried out GSEA of an in-house transcriptional dataset of six pairs of PLX4032-resistant and sensitive cell lines. In resistant cells, we found the dysregulation of lipid metabolism Hallmark gene sets “Cholesterol Homeostasis” and “Fatty Acid Metabolism” (Figure 1A; Supplementary Table S1). Several genes controlling main nodes of these pathways were modulated in the resistant cells and contributed to the enrichment of the gene sets, including for example, FASN, elongase ELOVL5, MVK, HMGCR, desaturase FADS2, Acyl-CoA Synthetase (ACSS2), ACAT2, DHCR24 and DHCR7 and Farnesyl Diphosphate Synthase (FDPS) involved in fatty acid metabolism and cholesterol homeostasis (Supplementary Figure S1).

The association between an altered metabolism and the BRAFi-resistant phenotype was evident also by the analysis carried out with the Metabolizer software, a tool allowing the identification of modular architecture of metabolic pathways using transcriptomic data, and highlighting pathway metabolic module activities relevant to a specific phenotype. The analysis of the transcriptomic profiles of the six pairs of PLX4032-resistant and sensitive cell lines identified three modules showing a significant (*p* < 0.05) reduction in the resistant cells. In addition, the metabolic modules “C5 isoprenoid biosynthesis, mevalonate pathway”, “FA biosynthesis and elongation” and “Cholesterol biosynthesis” associated to the pathways of terpenoid, FA and steroid synthesis showed a trend of modulation (*p* ≤ 0.1) (Supplementary Table S2).

### Lipid Profiles Associated With BRAFi Resistance

Given that lipid metabolic reprogramming appeared to be associated with the BRAFi-resistant phenotype, we examined if it was also accompanied by an alteration of lipid composition in the resistant cell lines. In a first set of experiments, we evaluated the total FA profile of three matched resistant and sensitive cell lines, selected based on their marked BRAFi-resistant phenotype (Vergani et al., 2022). Experiments were performed after 24 h cell culture in the absence of serum, in order to avoid possible confounding effects by FBS lipids. GLC analysis revealed a different composition between resistant and sensitive cells (Table 1). In fact, beyond differences in single FA, when these were categorized into 0 ones, resistant cells showed a general percent increase in MUFA or in PUFA, paralleled by a concomitant decrease in SFA (Figure 1B). The results obtained by the analysis of TG mass and composition cells mirrored what found in total lipid extract: also in this lipid subclass, resistant cells displayed an increase in MUFA counterbalanced by a decrease in SFA (Figure 1C). Noteworthy, resistant cells presented roughly an up to 3.5-fold reduction in TG mass compared to sensitive ones (Figure 1D). In terms of FA composition, the same conclusions can be drawn from CE analysis (Figures 1C,D, Supplementary Table S3), showing the 8% increase in MUFA in resistant cells counterbalanced by a similar decrease in SFA compared to sensitive cells. Interestingly, oleic acid (18:1) significantly contributes to this phenomenon, in keeping with oleic acid representing the preferred substrate of SOAT enzyme catalyzing esterification. Regarding total mass, while that of free cholesterol (FC) did not significantly change (data not shown), that of CE was dramatically reduced (up to 5-fold) in resistant cells (Figure 1D). Altogether, these results further suggested a possible association between an altered cellular lipid composition and BRAFi resistance in melanoma cells.

**TABLE 1:** Relative percentage of total FA detected by GLC in resistant LM16R, LM47R and LM36R cells compared to their sensitive parental counterparts.

Total FA (%)	LM16		LM16R		*p* value	LM47		LM47R		*p* value	LM36		LM36R		*p* value
	Mean	SD	Mean	SD		Mean	SD	Mean	SD		Mean	SD	Mean	SD	
14.0	5.47	0.40	2.31	0.22	0.01	2.76	0.29	2.30	0.34	ns	1.67	0.40	1.69	0.23	0.0005
16.1	9.00	0.06	6.09	0.15	0.001	3.51	0.01	7.21	0.49	0.008	7.02	1.03	6.15	1.10	ns
16.0	30.00	1.14	27.81	0.52	ns	30.42	1.24	25.92	1.63	ns	23.89	1.69	24.74	1.23	ns
16.0 DMA	2.82	0.68	3.56	0.35	ns	2.41	0.16	3.01	0.33	ns	3.98	0.12	3.20	0.27	ns
18.2	0.56	0.28	0.76	0.17	ns	4.62	0.10	0.97	0.37	0.005	1.40	0.52	1.51	0.61	ns
18.1	38.51	0.16	40.19	0.06	0.005	30.47	1.92	38.75	0.36	0.02	38.64	0.65	37.47	0.73	ns
18.0	5.21	0.90	6.21	0.02	ns	13.86	0.83	10.05	1.28	ns	10.51	0.37	12.30	0.37	0.03
18.1 DMA	0.65	0.18	0.56	0.03	0.03	0.28	0.07	0.52	0.12	ns	0.88	0.20	0.53	0.07	ns
18.0 DMA	1.01	0.25	1.80	0.05	0.03	1.95	0.38	1.77	0.03	ns	1.65	0.19	1.16	0.28	ns
20.4 w6	1.24	0.21	2.20	0.18	0.03	1.96	0.12	2.34	0.59	ns	2.92	0.40	3.27	0.44	ns
20.2	0.84	0.09	1.00	0.00	ns	0.82	0.29	1.04	0.05	ns	0.84	0.22	0.69	0.18	ns
20.1	1.05	0.03	0.75	0.02	0.007	0.40	0.12	0.60	0.04	ns	0.93	0.10	0.79	0.09	ns
20.0	0.20	0.04	0.23	0.02	ns	0.49	0.10	0.28	0.03	ns	0.17	0.10	0.17	0.13	ns
22.6	0.54	0.64	1.35	0.51	ns	2.23	0.49	2.05	0.12	ns	1.98	0.22	2.27	0.11	ns
22.0	0.73	0.08	1.05	0.22	ns	1.69	0.05	0.98	0.21	0.04	1.08	0.53	1.15	0.59	ns
24.1	1.54	0.18	2.12	0.12	ns	0.59	0.04	0.93	0.11	ns	1.32	0.20	1.37	0.16	ns
24.0	0.63	0.04	2.02	0.01	0.0004	1.54	0.13	1.28	0.13	ns	1.12	0.30	1.52	0.19	ns
SFA	42.24	0.5	39.62	0.9	ns	50.75	2.2	40.81	0.7	0.02	38.44	2.00	41.58	1.84	ns
MUFA	50.09	0.3	49.15	0.02	0.05	34.97	1.7	47.49	0.06	0.009	47.91	1.61	45.78	1.70	ns
PUFA	3.19	0.2	5.31	0.5	0.03	9.64	0.2	6.41	1.04	0.05	7.14	1.07	7.75	1.02	ns

Data obtained by calculating the % of each FA relatively to the total of SFA + MUFA + PUFA as 100% in melanoma cells. Mean values and standard deviations (SD) are reported *p* values by Student’s t test.

### Effects of Lipid Starvation on BRAFi Sensitivity

**FIGURE 2:**
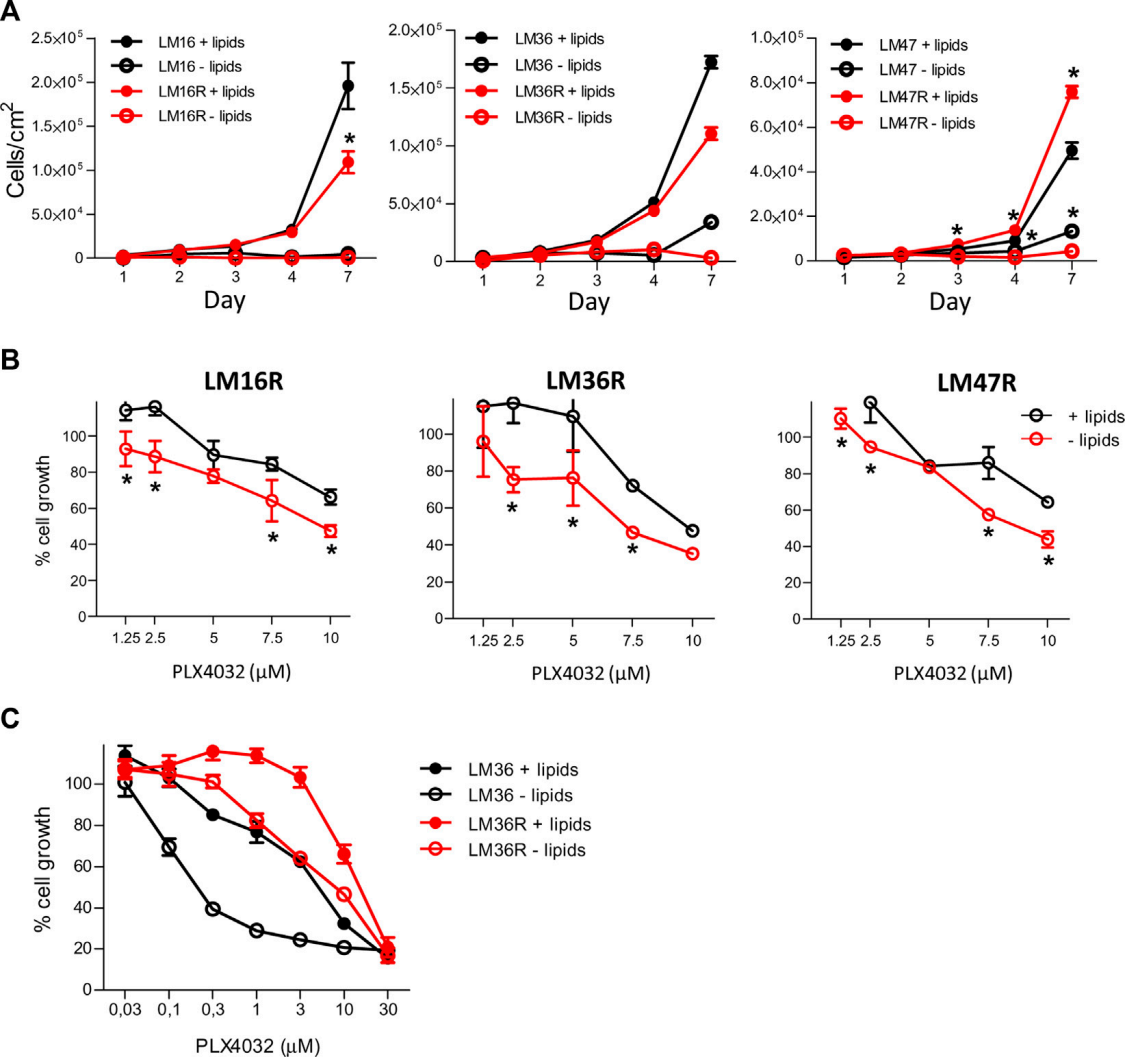
Effect of lipid starvation on the growth of melanoma cells and on PLX4032 sensitivity. **(A)**. Cells were seeded at equal density (8 × 10^3^ cells/well) in standard culture conditions. After 24 h, medium was replaced with lipid-free medium and cell growth was followed over time. Cells were counted using a Coulter Counter at the indicated time points. **(B)**. Dose-response analysis by CCK8 assay after 48 h treatment with PLX4032 in standard medium and lipid-free culture conditions after having seeded cells at the density of 12 × 10^3^ cells/well. In **(A)** and (B)_*_ *p* values < 0.05 by two-way ANOVA followed by Bonferroni correction. **(C)**. Cell growth as detected by CCK8 assay in LM36/LM36R cells after 48 h treatment with different concentrations of PLX4032, and in presence or absence of lipids. The IC_50_ values were as follows: 4.5 and 14.6 μM for LM36 and LM36R plus lipids, and 0.37 and 6.7 μM respectively in lipid-free condition. All experiments were repeated three times and a representative experiment is shown.

We hypothesized that the deregulation of lipid metabolism pathways may contribute to melanoma cell resistance and we examined the dependence of melanoma cell growth on extracellular lipids using pairs of PLX4032-resistant and sensitive cell lines and lipid-free culture conditions. Lipid starvation resulted in a time-dependent decrease of cell growth, less evident at high cell density, and more evident in resistant cells (Figure 2A). In fact, when we examined the ratio between the number of cells in standard medium versus the number of cells in lipid depleted medium after 7 days of growth, the effects of lipid deprivation appeared to be higher in drug-resistant cells than in the sensitive counterparts, because the extent of growth change was always higher in resistant cells, with 112.45 in LM16R versus 37.8 in LM16, 34.43 in LM36R versus 5.0 in LM36 cells, and 17.5 in LM47R versus 3.7 in LM47 cells. Cell growth inhibition assays indicated that cells grown in the absence of lipids were more sensitive to PLX4032 than when grown under standard culture conditions, independently of their respective sensitivity/resistance (Figures 2B,C). Given that the curves are mostly parallel, the shift in sensitivity can be easily appreciated. PLX4032 showed about 1.5-fold increased efficacy upon lipid starvation as shown by a comparison of IC_30_ values (Supplementary Table S4).

### Dysregulation of Lipid Metabolism-Associated Genes in BRAFi Resistant Melanoma Cells

**FIGURE 3:**
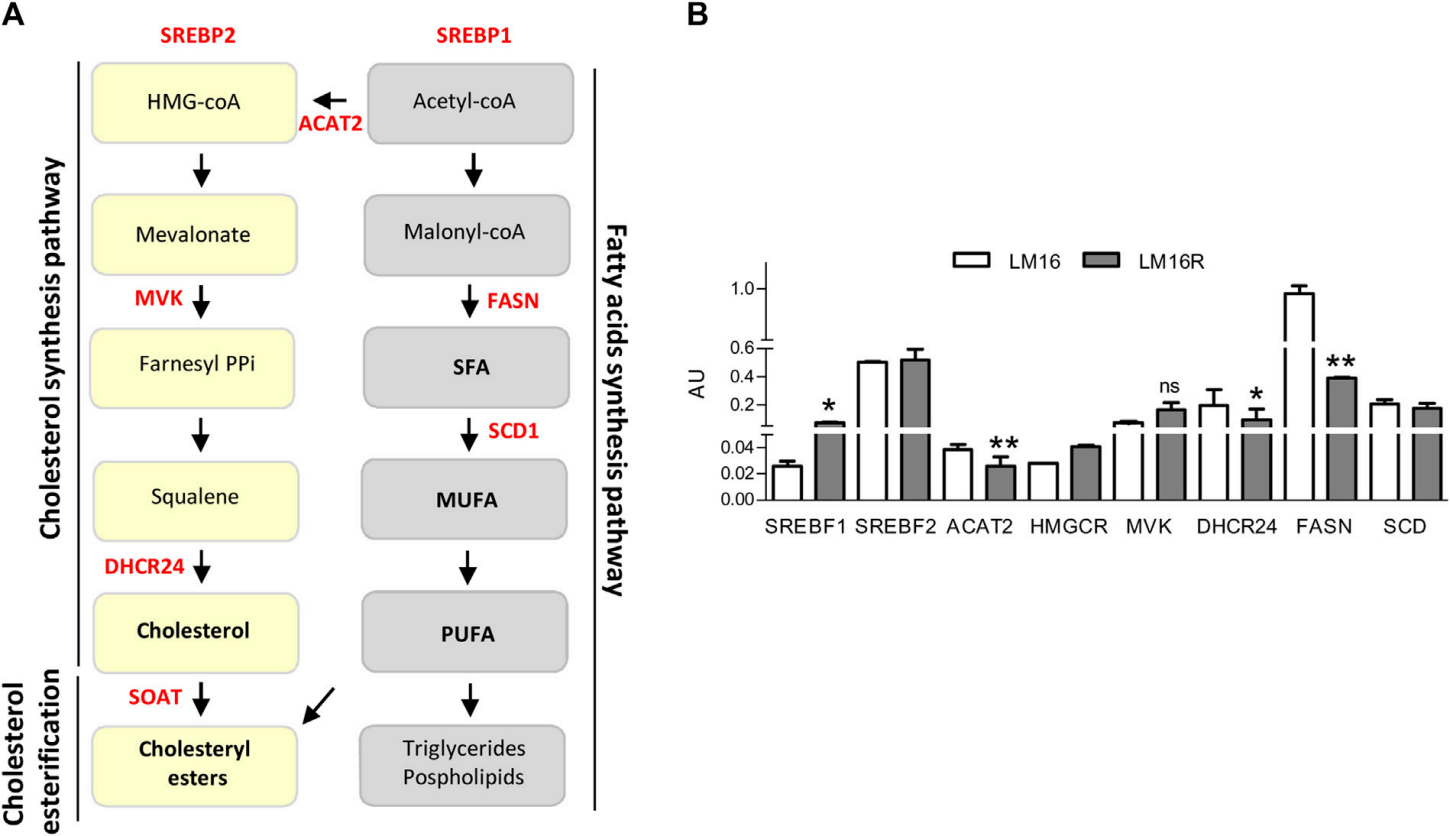
Expression of lipid metabolism genes in LM16 and LM16R cell lines. **(A)**. Cholesterol and fatty acids synthesis pathways. Genes of interest are highlighted in red. **(B)**. Expression levels of lipid metabolism-related genes in LM16R compared to LM16 cell line as evaluated by qRT-PCR. AU, Arbitrary Units. *p < 0.05; **p < 0.01 by unpaired Student’s t test. Determinations were repeated three times. ns: not significant.

Based on the results obtained from GSEA and metabolizer, a set of genes encoding for enzymes controlling crucial nodes of FA pathways and cholesterol synthesis, i.e., FASN, SCD, Sterol Regulatory Element Binding Transcription Factor 1 (SREBF1) and 2 (SREBF2), ACAT2, HMGCR, MVK, and DHCR24 was selected for analysis in LM16R compared to LM16 cells (Figure 3A). The gene expression levels of ACAT2, DHCR24 and FASN resulted significantly downregulated in the resistant cells compared to the sensitive counterparts, while SREBF1 and HMGCR show a significant increase and no changes were observed for MVK, SREBF2 and SCD (Figure 3B).

**FIGURE 4:**
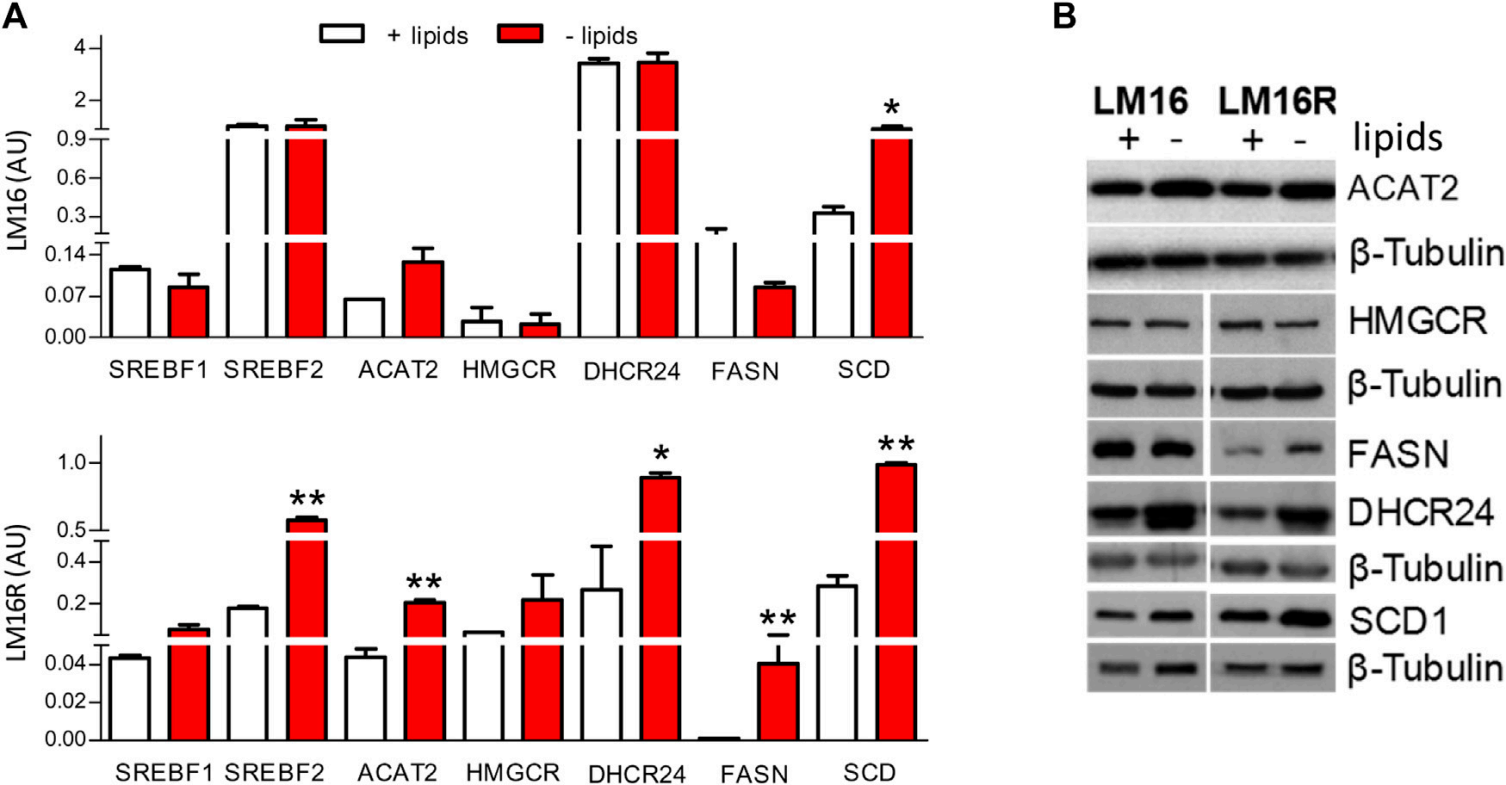
Effects of lipid starvation on cholesterol and fatty acid synthesis pathways in LM16 and LM16R cell lines. **(A)**. Expression of lipid metabolism-related genes evaluated by qRT-PCR in standard and lipid-free condition. AU, Arbitrary Units. *p < 0.05; **p < 0.01 by unpaired Student’s t test. **(B)**. Expression of lipid metabolism-related enzymes as evaluated by western blot upon lipid starvation for 48 h β-tubulin was used as loading control. Determinations were repeated two/three times.

Since the dysregulation of lipid metabolism genes could be relevant for BRAFi resistance, we evaluated the effect of extracellular lipid deprivation on the expression levels of the selected genes. Compared to standard culture conditions, lipid-free medium induced the expression of SREBF2, ACAT2, DHCR24, and FASN genes in resistant cells (Figure 4A). In addition, lipid starvation upregulated the expression of the SCD transcript, previously involved in melanoma BRAFi resistance and regulated by Melanocyte Inducing Transcription Factor (MITF) (Pisanu et al., 2018; Vivas-García et al., 2020). The induction of ACAT2, FASN, DHCR24, and SCD1 upon lipid deprivation was evident at the protein level. In contrast, lipid starvation resulted in the lack of relevant modulation of HMGCR (Figure 4B) and in slight reduction of the levels of the unprocessed precursor form of SREBP1 and SREBP2 (not shown). The comparison of gene modulations induced by lipid deprivation between resistant and parental cell lines evidenced a higher modulation in resistant cells (Supplementary Material Western blot images), suggesting a stronger effect on lipid biosynthetic pathways.

### ACAT2 Targeting Impacts on BRAFi Resistance in Melanoma Cells

**FIGURE 5:**
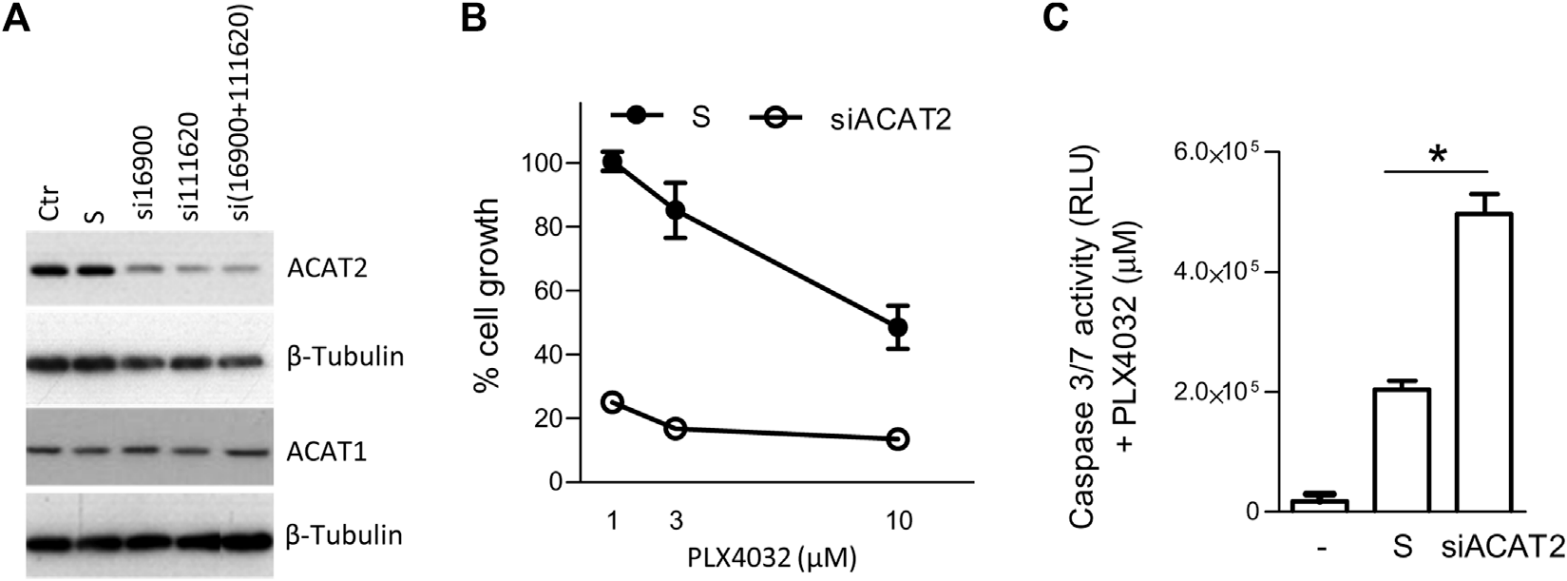
ACAT2 downregulation impairs BRAFi resistance in melanoma cell lines. **(A)**. LM16R cells were transfected with two ACAT2-directed siRNAs (si16900 and si111620) or with their mixture in standard culture conditions. The expression levels of ACAT2 and ACAT1 were evaluated by western blotting 72 h after transfection. β-tubulin was used as loading control. **(B)**. Effects of ACAT2 siRNA mixture (72 h) on PLX4032 sensitivity determined by CCK8 assay. **(C)**. Effects of ACAT2 siRNA (72 h) on PLX4032 (10 µM) sensitivity by caspase 3/7 activation assay. -: untreated control; S: scrambled control; siACAT2: ACAT2 siRNA mixture. *p < 0.05 by one-way ANOVA followed by Bonferroni correction. The experiment was repeated three times.

The relevance of SCD1, MVK, FASN and DHCR24 in melanoma progression or drug resistance is already supported by our previous studies and by the literature (Pisanu et al., 2018; Theodosakis et al., 2019; Stamatakos et al., 2021). Considering ACAT2 dysregulation, both at gene and protein level, in resistant cells compared to the sensitive ones, and in lipid starvation compared to standard culture conditions, we evaluated the effects of ACAT2 targeting on overcoming BRAFi resistance. To test whether the sensitivity to BRAFi in resistant cells would increase by reducing ACAT2 levels, we silenced ACAT2 in LM16R cells using specific siRNAs. Reduced levels of ACAT2 did not impact on cell proliferation while resulted in increased sensitivity to PLX4032 (Figure 5A,B) an effect associated with the increased activation of caspase 3/7 (Figure 5C).

### SOAT Targeting by Avasimibe Potentiates the Effects of PLX4032 in Resistant Melanoma Cells

**FIGURE 6:**
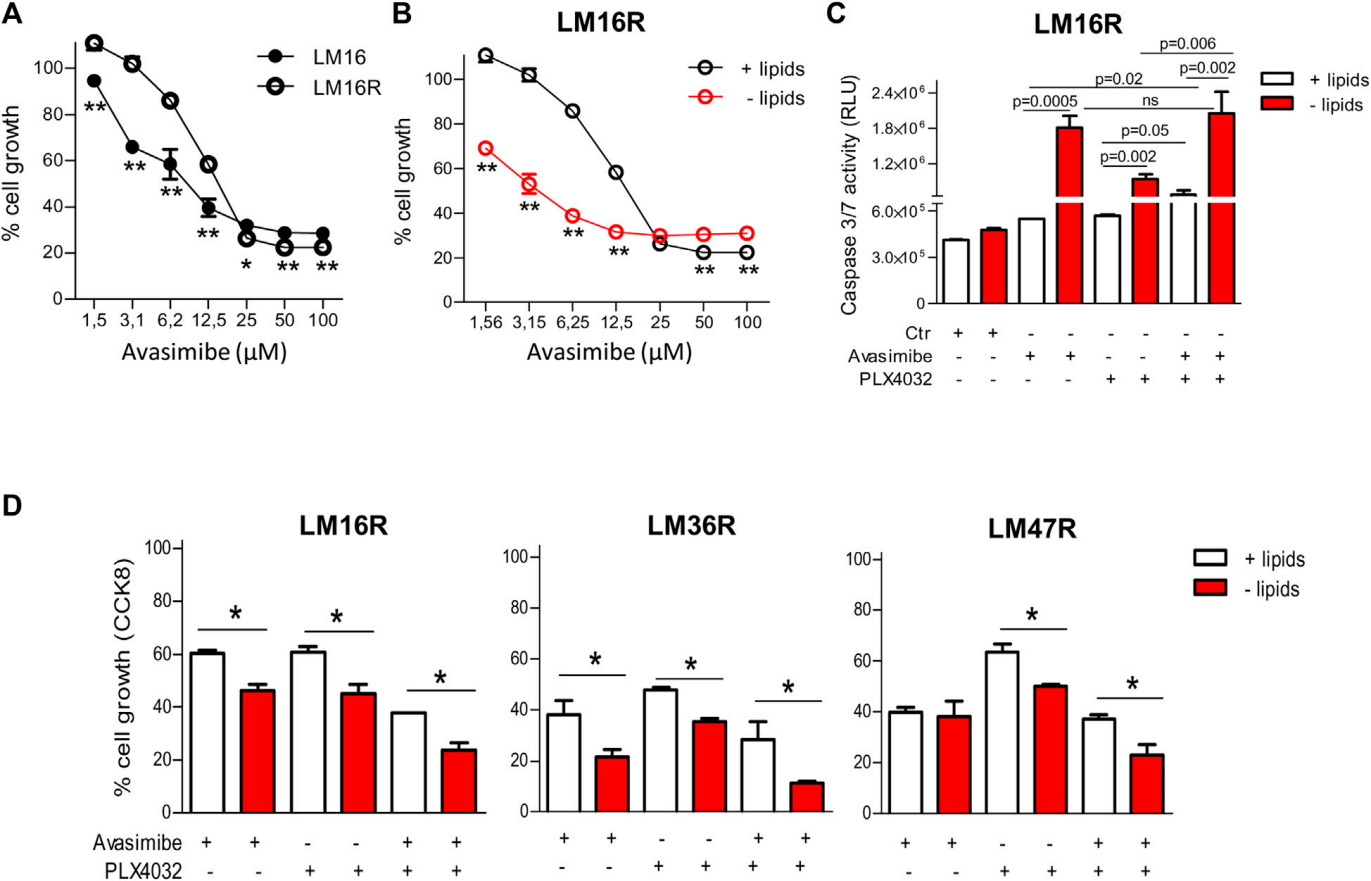
Antiproliferative effects of avasimibe and of the combined treatment with PLX4032 in melanoma cell lines. **(A)**. Sensitivity of LM16/LM16R cells to avasimibe in standard culture conditions. IC_50_ values of avasimibe for LM16R and LM16 parental cells were 17.1 and 11.1 µM in standard culture medium. **(B)**. Sensitivity of LM16R cells to avasimibe in standard culture condition and upon lipid starvation. IC_50_ values of avasimibe were 17.1 µM in standard condition and 4.2 µM in lipid-free medium. In **(A)** and **(B)** **p < 0.01 by Two-way ANOVA followed by Bonferroni correction. **(C)**. Apoptosis induction after 48 h treatment with avasimibe (12.5 µM) and PLX4032 (3 µM) and with the combination in standard and lipid-free medium as detected by caspase 3/7 activity. *p* values by unpaired Student’s t test. ns: not significant. Ctr: untreated control. **(D)**. Sensitivity of LM16R, LM47R and LM36R cells to avasimibe (25 µM), PLX4032 (3 µM) alone or in combination as determined by CCK8 assay. Data are expressed as percent of cell growth compared to untreated control. *p < 0.05 by One-way ANOVA followed by Bonferroni correction. Experiments were repeated four times.

Based on the findings of altered CE category in resistant cells and taking advantage of the clinically available SOAT inhibitor avasimibe, we assessed the importance of SOAT in sustaining cell growth. Cell treatment with avasimibe determined a slightly lower inhibitory effect in LM16R compared to LM16 cells (Figure 6A; Supplementary Figure S2A) but, compared to standard culture conditions, a 4-fold increased potency of avasimibe was observed in LM16R cells under lipid starvation (Figure 6B). Treatment with avasimibe significantly induced caspase 3/7 activity in resistant cells, an effect further enhanced in lipid starvation conditions, thus confirming the dependence on lipid synthesis pathways in BRAFi-resistant cells (first two columns, Figure 6C; Supplementary Figure S2B).

To define whether avasimibe could improve the efficacy of PLX4032, drug combination studies were carried out using a simultaneous 48 h combination exposure with two concentrations of PLX4032 (3 and 10 µM) and of avasimibe (12.5 and 25 µM), in the presence or absence of lipids (Table 2). We found that the drug combination tends to display a synergistic effect (CI < 1), and that, under selected conditions, lipid deprivation improved the synergistic interaction between PLX4032 and avasimibe in the BRAFi-resistant variants LM16R, LM36R and LM47R (Figure 6D). Specifically, synergism was observed in all the cell lines for exposure to 3 µM PLX4032 irrespectively of avasimibe concentration, while the combination with 10 µM PLX4032 resulted in lack of synergistic interaction, except than for LM36R cells. The synergistic interaction between PLX4032 (3 µM) and avasimibe (12.5 µM) was associated with increased caspase 3/7 activation in LM16R cells cultured in lipid-free medium (Figure 6C).

**TABLE 2:** Analysis of the drug interaction between PLX4032 and avasimibe in LM16R, LM36R and LM47R cells^a^.

CI^b^						
	LM16R		LM36R		LM47R	
	+lipids	−lipids	+lipids	−lipids	+lipids	−lipids
10 µM PLX4032	1.36 ± 0.15	0.89 ± 0.25	1.02 ± 0.35	0.82 ± 0.17	1.13 ± 0.36	0.91 ± 0.25
25 µM avasimibe						
3 µM PLX4032	1.15 ± 0.33	0.75 ± 0.29	0.88 ± 0.25	0.78 ± 0.18	1.03 ± 0.33	0.87 ± 0.24
25 µM avasimibe						
10 µM PLX4032	1.24 ± 0.35	0.81 ± 0.16	0.5 ± 0.199	0.44 ± 0.0035	1.15 ± 0.48	1.25 ± 0.45
12.5 µM avasimibe						
3 µM PLX4032	0.87 ± 0.40	0.69 ± 0.29	0.58 ± 0.13	0.42 ± 0.027	0.9 ± 0.27	0.61 ± 0.26
12.5 µM avasimibe						

a Cell sensitivity was assessed by cell growth inhibition assay. Cells were seeded and 24 h later exposed to each drug and to their simultaneous combination for 48 h in presence or absence of lipids; cells were then counted using a cell counter.

b CI: combination index. The drug interaction was analyzed by the Chou and Talalay method, by calculating a CI. CI values indicating synergistic drug interactions are in bold. Mean CI values **±** SE of three independent experiments are reported.

### Effect of PLX4032 Exposure and Lipid Starvation on Malondialdehyde and Ferroptosis Induction

**FIGURE 7:**
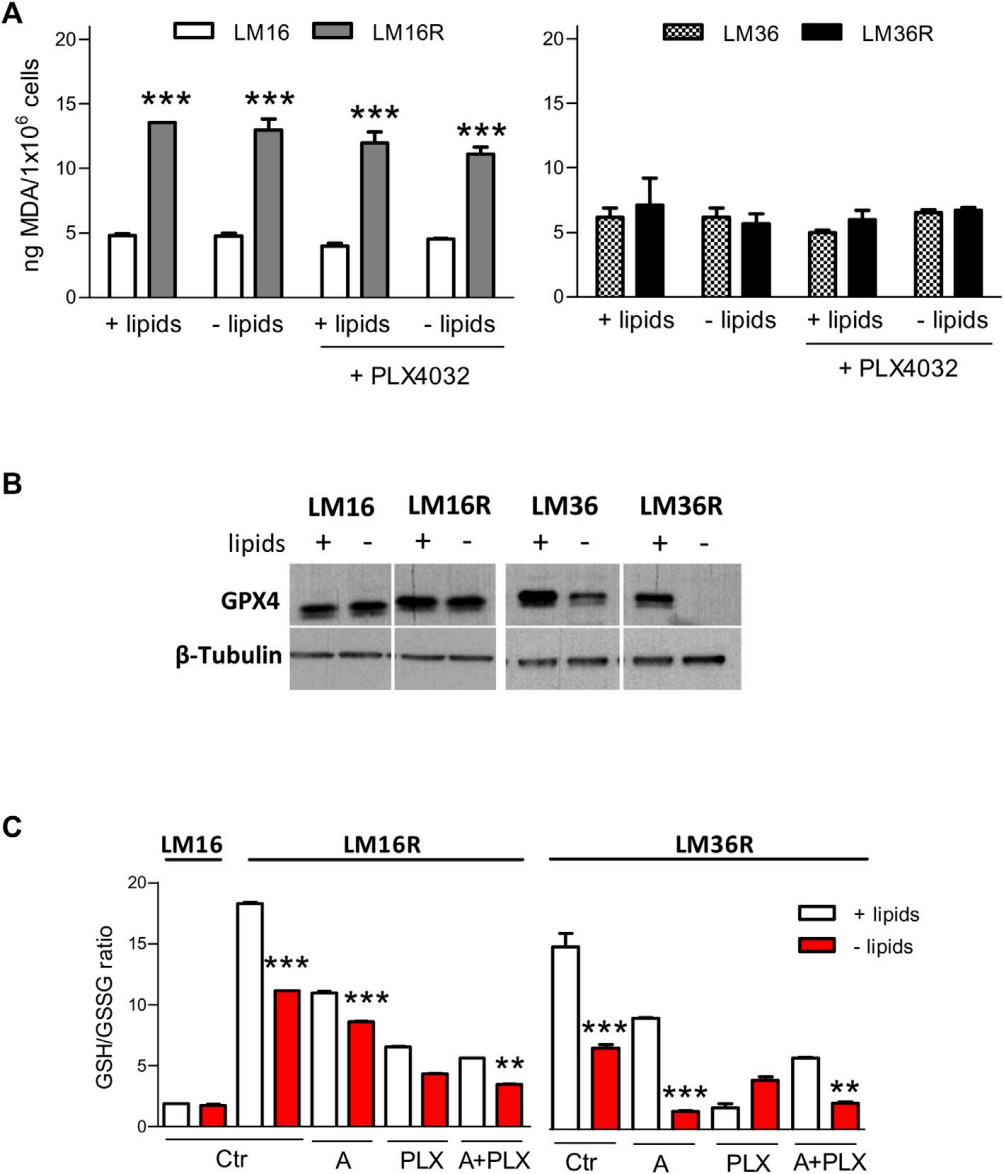
Effects of lipid starvation on ferroptosis in melanoma cell lines. **(A)**. Lipid peroxidation is unaffected by lipid starvation in melanoma cells. Induction of lipid peroxidation as detected by MDA measurement after 24 h culture in standard or in lipid-free medium and after treatment for 24 h with PLX4032 at concentrations corresponding to IC_80_ as calculated after 72 h exposure (0.3 and 0.89 µM for LM16 and LM36, respectively; 23 and 13.9 µM for LM16R and LM36R, respectively). ***p < 0.0001 by one-way ANOVA followed by Bonferroni correction. Experiments were repeated twice. **(B)**. GPX4 expression levels in lysates obtained from LM16, LM16R, LM36, LM36R cells grown for 48 h in the presence or absence of lipids. β–tubulin was used as loading control. **(C)**. Induction of ferroptosis as detected by reduced GSH/GSSG ratio at 24 h culture in standard or in lipid-free medium after treatment with PLX4032 (3 µM), avasimibe (12.5 µM), alone or in combination. Ctr: untreated control; A: avasimibe; PLX: PLX4032; A + PLX: avasimibe + PLX4032. **p < 0.01, ***p < 0.0001 by one-way ANOVA followed by Bonferroni correction.

To gain insights into the relevant mechanisms of cell response upon lipid deprivation, we examined lipid peroxidation and ferroptosis. The influence of lipids and PLX4032 on the levels of the lipid peroxidation marker MDA was evaluated in LM16/LM16R and LM36/LM36R cell line pairs. Cells were exposed for 24 h to PLX4032 at concentrations corresponding to the IC_80_ at 72 h exposure, in the absence or presence of lipids. LM16R cells showed about 3-fold higher levels of MDA compared to LM16 cells (Figure 7A). No changes were observed in LM16 and LM16R cells following lipid deprivation or PLX4032 exposure. Conversely, LM36 and LM36R showed similar basal levels of MDA and no variations were observed after lipid deprivation or PLX4032 exposure. The possible induction of ferroptosis was then analyzed in LM36R cells, where lipid deprivation reduced the levels of GPX4, an inhibitor of ferroptosis, and in LM16R cells, in which GPX4 resulted unaffected by lipid starvation (Figure 7B). Since GPX4 is an enzyme that uses GSH as a substrate to block ROS production, we analyzed GSH/GSSG ratio, that is modulated by the induction of ferroptosis. A reduced GSH/GSSG ratio was evident both in LM16R and LM36R cells upon lipid starvation or treatment with avasimibe and PLX4032, used alone or in combination at synergistic concentrations (Figure 7C). Notably, lipid starvation increased ferroptosis in LM16R cells but not in the parental LM16 cells.

## Discussion

Metabolic reprogramming represents one of the hallmarks of cancer cells exploited in the field of research on drug resistance. In melanoma, metabolic phenotypes have been linked both to activated RAS-RAF-MAPK and PI3K/AKT intrinsic oncogenic signaling in tumor cells and to interactions within the tumor microenvironment (Fischer et al., 2018). Although the melanoma key oncogenic pathways typically promote glycolysis, alternative pathways including FA oxidation become essential in nutrient constraints due to inadequate vascularization, and metabolic flexibility provides a survival advantage to tumor cells (Pellerin et al., 2020). Metabolic reprogramming contributes to melanoma heterogeneity and progression, and appears to be implicated in resistance to targeted therapy (Hernandez-Davies et al., 2015; Viswanathan et al., 2017; Aloia et al., 2019). Among the most prominent metabolic reprogramming features, there is an increased rate of lipid synthesis due to the activation of the lipogenic pathway, and *de novo* FA biosynthesis, which have been associated to drug resistance in cellular models (Pisanu et al., 2018; Talebi et al., 2018; Garandeau et al., 2019; Delgado-Goni et al., 2020). In addition, different studies have demonstrated a role for lipid uptake in melanoma progression, through the lipid transfer by stromal adipocytes, or adipocyte-secreted exosomes carrying substrates and proteins that fuel FA oxidation and melanoma cell metabolism (Lazar et al., 2016; Zhang et al., 2018). Melanoma cells can take up lipids from aged fibroblasts, via Fatty acid transport protein 2, and use them to resist targeted therapy (Alicia et al., 2020). Moreover, it has been reported that a high-fat ketogenic diet enhances growth of BRAF V600E melanoma cells xenotransplanted into mice and that hypolipidemic agents can constrain tumor growth (Xia et al., 2017).

In the present study, the analysis of the transcriptomic profiles and lipid composition in melanoma resistant cells highlighted dysregulation of lipid metabolism involving FA and cholesterol, as well as alterations in the lipid composition. In fact, according with gene expression data, when considering lipid mass, we found profound decreases in TG and CE mass (respectively 3.5 and 5 fold) in resistant compared to sensitive cells. The lack of difference on FC mass is possibly ascribable to the short timing of our experimental conditions compared to cell cholesterol turnover, and further studies by incorporation of radiolabeled acetate into cholesterol are necessary to evaluate the potential differential rate in cholesterol biosynthesis in resistant cell lines. The increased percentage of incorporation of oleic acid into CE in resistant cells indirectly proves the alteration of the machinery of cholesterol esterification, since it is known that in most cell types the monounsaturated oleic acid represents the preferential substrate for SOAT (Seo et al., 2001). Gene expression data also indicated perturbations in desaturases and elongases (Supplementary Figure S1), such as SCD1, which converts palmitic and stearic SFA in palmitoleic and oleic MUFA. This evidence is corroborated by the percent increase in MUFA and PUFA not only in total lipid extract profile, but also in CE and TG. Moreover, a trend of percent increase of FA typical of sphingomyelins (C20:0, C20:1, C20:2, C22:0, C24:0 and C24.1) was also evident in resistant cell lines. Altogether, differences in saturation of FA may not only influence membrane fluidity but also membrane exchanges, enzyme activation and protein/protein interactions, thus altering cell behavior and metabolism at different levels.

These results defined the main nodes in the cholesterol and FA biosynthetic pathways potentially involved in determining the different lipid composition between sensitive and resistant cells. One example is represented by enhanced levels of the SCD1 enzyme, resulting in higher MUFA concentrations. The altered expression of enzymes regulating lipid metabolism such as FASN has been proposed to contribute to tumor resistance (Szlasa et al., 2020), and we previously showed that molecular targeting of FASN in melanoma resistant cell lines increased the sensitivity to the BRAFi PLX4032 and resulted in enhanced expression of DHCR24 gene (Stamatakos et al., 2021). In addition, the HMGCR inhibitor simvastatin was shown to further inhibit cell proliferation when combined to vemurafenib (Theodosakis et al., 2019). All these approaches suggest that resistance could be counteracted by drug combinations with FA/cholesterol metabolism inhibitors.

Similarly, we show here that molecular silencing of ACAT2 impaired resistance to PLX4032, further supporting the evidence that interfering with cholesterol metabolism may impair melanoma cells BRAFi resistance. The enhanced ACAT2 expression observed in lipid-starved resistant melanoma cells could be ascribed to mechanisms involving SREBF1/2 expression, which control upstream lipid metabolism pathways, and display upregulation in resistant cells.

Genes selected for encoding critical enzymes involved in lipid metabolism pathways showed altered expression level in resistant cells, further sustaining the role of altered lipid metabolism in the BRAFi-resistant phenotype. The effect of deregulated lipid metabolism in resistant cells was evidenced upon lipid starvation, a condition modulating the expression of genes like SREBF2, ACAT2, DHCR24, FASN and SCD. Despite lipid deprivation resulted in increased SREBF mRNA levels, a slight reduction of unprocessed precursor SREBF1 and SREBF2 protein expression was observed in LM16R and LM36R cells. This finding is consistent with the hypothesis that an increase of the processing of the precursors in resistant cells could result in the augmentation of the active form of the enzymes. In this context, Talebi and colleagues (Talebi et al., 2018) have shown that BRAFi-sensitive cells respond to BRAF inhibition down-regulating the processing of SREBP1 and thereby lipogenesis, a process, that is, restored in resistant cells to protect from reactive-oxygen species-induced damage and lipid peroxidation. The discrepancy with our results may reside in the different conditions tested in our study, where basal expression and expression in lipid-free medium were compared. Indeed, down-regulation of lipid metabolism genes was observed under standard culture conditions in resistant cells, whereas lipid deprivation induced the expression of lipid metabolism genes. In this regard, when comparing our results with those shown by Talebi et al., the key point may be the type of SREBP1 down-regulation, particularly the concomitant downregulation of the precursor and active form evidenced by Talebi in sensitive cells, or the downregulation of the precursor found by us speculatively associated to the expression of the active form.

An interesting finding of this study is that drug-resistant cells become more sensitive to BRAFi following lipid deprivation. Such a sensitization was more evident when cells were treated with the combination of PLX4032 and the SOAT inhibitor avasimibe, thereby supporting that metabolic changes can reveal cell vulnerabilities. According to its primary target, the SOAT-inhibitor avasimibe displayed a lower inhibitory effect in LM16R compared to LM16 cells, thus highlighting the importance of SOAT in sustaining cell growth. To confirm the importance of lipid metabolism in tumor growth, a 4-fold increased potency of avasimibe was observed in LM16R cells under lipid starvation compared to standard culture condition.

A major goal in melanoma resistance studies is the identification of novel targets for combination therapy in an attempt to overcome resistance to BRAFi. In this context, SOAT represented a additional target to be tested for its role in the synthesis of CE, which displayed alterations in resistant cells. SOAT, a membrane-bound enzyme that catalyzes the biosynthesis of CE from long-chain fatty acyl-CoA and cholesterol, is a key enzyme involved in the control of cholesterol storage. SOAT activity prevents unnecessary FC within the cell membrane by the cholesterol esterification and storage of CE as lipid droplets within the cell (Rogers et al., 2015). The intracellular esterification of FC determines its solubility in the cell membrane lipids, preventing the toxic accumulation of FC in cell membrane fractions (Chang et al., 1997). Furthermore, alterations in cholesterol homeostasis may interfere with lipid rafts, membrane microdomains which regulate the assembly and functioning of cell signaling pathways, including growth, adhesion, migration, invasion and apoptosis (Vona et al., 2021).

Avasimibe, which we combined with PLX4032 in melanoma cell lines, was extensively studied for its anti-hyperlipidemic and anti-atherosclerotic effects (Giovannoni et al., 2003). Avasimibe was reported to reduce cholesterol level, macrophage infiltration, and the expression and activity of matrix metalloproteinase, thus limiting the risk of atherosclerosis in clinical studies (Rodriguez and Usher, 2002).

Recent studies reported the *in vitro* antitumor effects of avasimibe on a variety of human tumor cells, by the reduction of CE storage in lipid droplets and increased of intracellular FC levels leading to apoptosis and suppression of cell proliferation (Lee et al., 2015; Gao et al., 2021). In glioma cell lines, avasimibe showed inhibition of cell proliferation, migration and invasion (Bemlih et al., 2010; Luo et al., 2020). In our cellular models, avasimibe ameliorated the antiproliferative effect of PLX4032, and increased caspase-3/7 activation and oxidative stress associated with ferroptosis. These effects were enhanced when the cells were cultured in lipid-free conditions. The reduction in GPX4 expression is not specific for LM36R cells. Though less evident, this feature is observed under lipid deprivation conditions in LM36, as well. Conversely, no variation in GPX4 expression is found following lipid deprivation in LM16 and LM16R cells. This could be dependent on the different molecular background of the two cell line pairs (Vergani et al., 2022). As far as ferroptosis is concerned, we hypothesized that cellular mechanisms not related to MDA levels are implicated. Indeed, Miao and colleagues (Miao et al., 2022) have shown that no changes in MDA levels occur following GPX4 knockdown. Therefore, we can conclude that at least two events associated with ferroptosis occur in LM36R cells upon shifting to lipid deprivation conditions, i.e., decrease of GSH/GSSG ratio (that is further decreased by treatment) and GPX4 downregulation. Ferroptosis has been shown to be modulated by exogenous MUFA (Magtanong et al., 2019) and by lipogenic pathways that modulate BRAFi resistance (Hong et al., 2021).

In keeping with our study, a clinical relevance of the metabolism deregulation in acquired resistance to BRAFi emerges from gene expression and survival data of publicly available datasets of melanoma patients. In fact, Delgado-Goni reported a dependence on lipid metabolism associated with higher PGE2 synthesis and Prostaglandin E synthase overexpression in BRAFi-resistant clones and in patients following progression to BRAFi-resistant disease (Delgado-Goni et al., 2020). Increased β-oxidation of FA was observed in BRAF/MEKi resistant cells and was correlated with poor clinical outcome (Shen et al., 2020). Several genes implicated in melanoma FA metabolism have been reported to correlate with the expression of MITF, a hallmark marker of the “proliferative to invasive” phenotype switch (Verfaillie et al., 2015), or of AXL, another hallmark marker of a de-differentiated and drug-resistant phenotype (Dugo et al., 2015). In addition, SCD expression positively correlated with that of MITF-regulated proliferative signature both in TCGA tumor samples and in the Cancer Cell Line Encyclopedia melanoma gene expression data (Vivas-García et al., 2020).

In conclusion, our study adds to the available literature evidencing that lipid metabolism entails molecular determinants of vulnerabilities of treatment resistant melanoma. Since systemic metabolism in cancer patients can be modulated by dietary interventions (Vernieri et al., 2022), this information may become exploitable in patients to improve the clinical efficacy of treatment with BRAFi.

## Data Availability

Publicly available dataset GSE201913 was analyzed in this study. This data can be found here: GEO repository https://www.ncbi.nlm.nih.gov/geo/query/acc.cgi.

## Abbreviations

ACAT/SOAT, acyl-CoA cholesterol acyl transferase; ACAT2, acetyl-CoA acetyltransferase 2; BRAFi, BRAF inhibitors; CCK8, cell counting kit 8; CE, cholesteryl esters; CI, combination index; DHCR24, 24-dehydrocholesterol reductase; ELOVL5, elongase of very-long fatty acid 5; FA, fatty acids; FASN, fatty acid synthase; FC, free cholesterol; FDPS, farnesyl diphosphate synthase; GLC, gas-liquid chromatography; GPX4, glutathione peroxidase 4; GSEA, gene set enrichment analysis; GSSG, glutathione disulfide (oxidized glutathione); GSH, reduced glutathione; HMGCR, 3-hydroxy-3-methylglutaryl-CoA reductase; MDA, malondialdehyde; MITF, melanocyte inducing transcription factor; MUFA, monounsaturated fatty acids; MVK, mevalonate kinase; NES, normalized enrichment score; PUFA, polyunsaturated fatty acids; SCD1, stearoyl–CoA desaturase 1; SFA, saturated fatty acids; siRNA, small interfering RNA; SREBF1/2, sterol regulatory element binding transcription factor 1/2; TG, triglycerides.
