# Supplementary material for: Targeting of the Lipid Metabolism Impairs Resistance to BRAF Kinase Inhibitor in Melanoma
Source: Front Cell Dev Biol. 2022 Jul 13;10:927118. doi: 10.3389/fcell.2022.927118 (PMC9326082; doi:10.3389/fcell.2022.927118)
Supplement: Supplementary file 1 [file DataSheet1.ZIP › Vergani E_revised supplementary material 10-6-22/Vergani E-Supplemental material western blot images final.pdf]

SUPPLEMENTARY MATERIAL WESTERN BLOT IMAGES

ACAT2

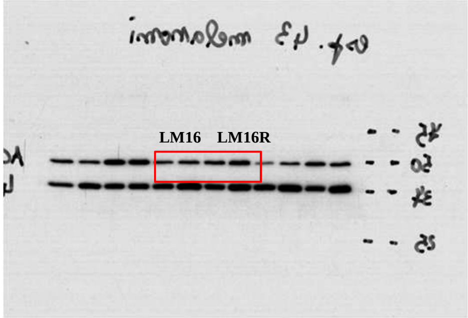

β-Tubulin

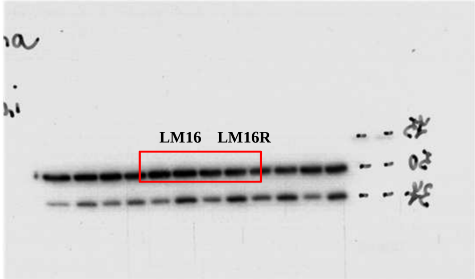

HMGCoA

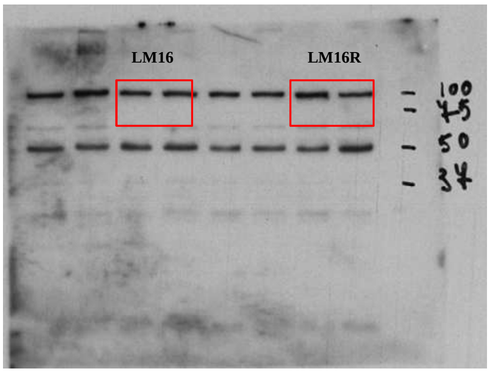

β-Tubulin

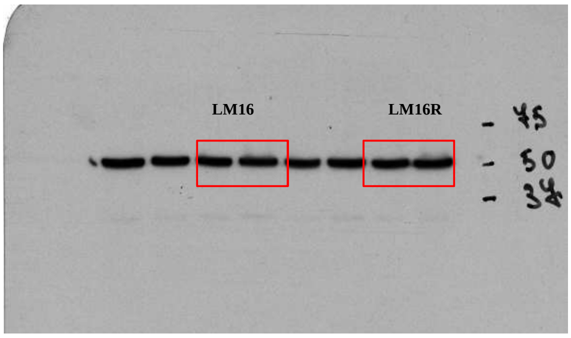

FASN

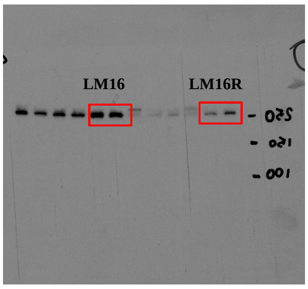

DHCR24

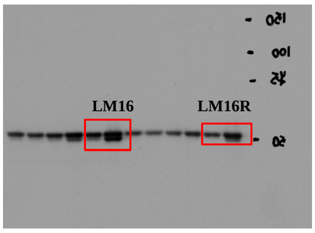

β-Tubulin

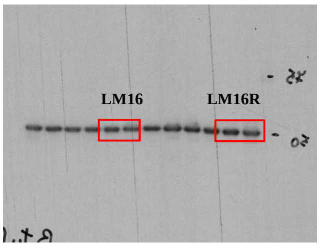

SCD1

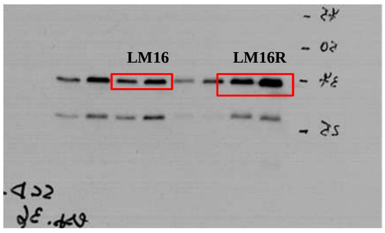

β-Tubulin

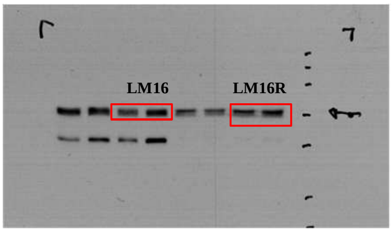

Western blot images for Figure 4

Condition	ACAT 2	Tubulin	Ratio
LM16 Standard	6398884	20787958	0,307816862
LM16 lipid depleted	8704244	22590045	0,385313265
LM16R Standard	9740025	20534133	0,474333394
LM16R lipid depleted	11398447	18971728	0,600812272
	HMGCoA R	Tubulin	Ratio
LM16 Standard	17620567	31694098	0,555957358
LM16 lipid depleted	20353963	35705520	0,570050877
LM16R Standard	19266436	35682406	0,539942178
LM16R lipid depleted	18312770	35000284	0,523217754
	FASN	Tubulin	Ratio
LM16 Standard	14648873	17552464	0,834576445
LM16 lipid depleted	13895185	22508856	0,6173208
LM16R Standard	5488952	19493675	0,28157605
LM16R lipid depleted	9993864	22062812	0,452973266
	DHCR24	Tubulin	Ratio
LM16 Standard	22281136	17552464	1,269402176
LM16 lipid depleted	34445947	22508856	1,53032864
LM16R Standard	17004119	19493675	0,872289037
LM16R lipid depleted	30147332	22062812	1,366431985
	SCD1	Tubulin	Ratio
LM16 Standard	10531957	10329922	1,019558231
LM16 lipid depleted	16033726	14578965	1,09978493
LM16R Standard	18276483	12125922	1,507224193
LM16R lipid depleted	29980760	13628804	2,199808582

Densitometric analysis was used to measure the band intensity. The relative expression levels (ratio between the band intensity of the protein of interest and the corresponding band intensity of tubulin) was determined by ImageQuant 5.2 software.

Western blot images for Figure 4

ACAT1

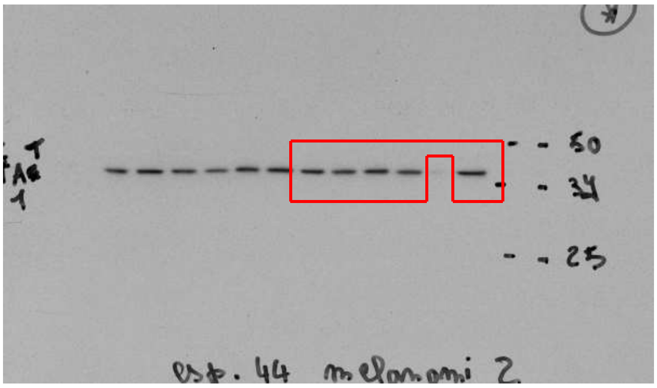

β-Tubulin

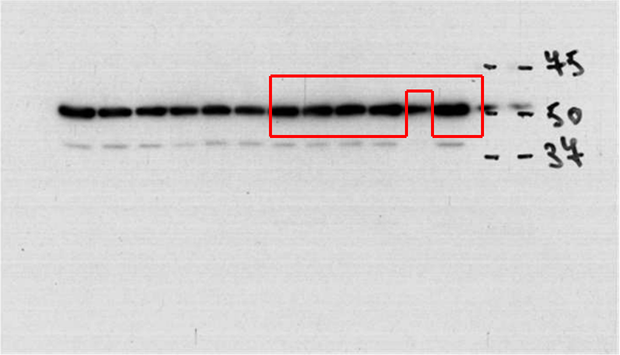

ACAT2

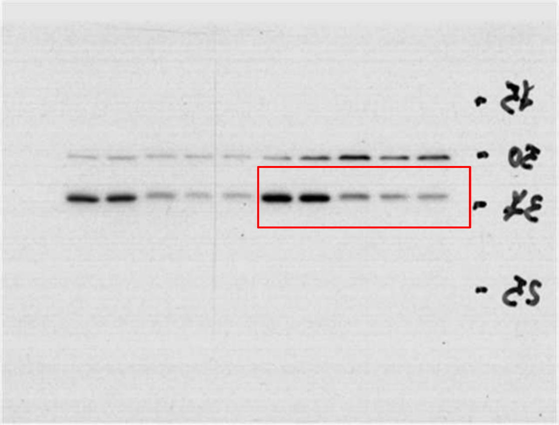

β-Tubulin

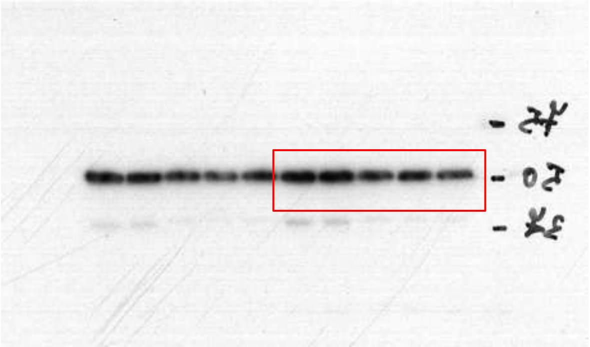

Western blot images for Figure 5

Condition	ACAT1	Tubulin	Ratio
Ctr	869343	1610109	0,539928
S	686118	1652299	0,415251
si16900	1114914	1852945	0,601698
si111620	1102296	2018999	0,545962
si16900+si111620	1579556	2793502	0,565439
	ACAT2	Tubulin	Ratio
Ctr	10092323	17628850	0,572489
S	9160176	17980835	0,509441
si16900	3447906	13470474	0,25596
si111620	2208134	14072720	0,156909
si16900+si111620	2027182	12782070	0,158596

Densitometric analysis was used to measure the band intensity. The relative expression levels (ratio between the band intensity of ACAT1 or ACAT2 and the corresponding band intensity of tubulin) was determined by ImageQuant 5.2 software.

Western blot images for Figure 5

LM16

GPX4

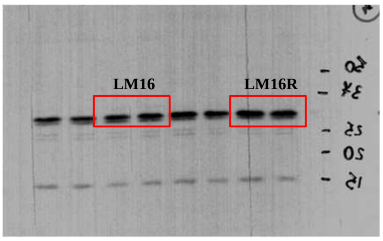

β-Tubulin

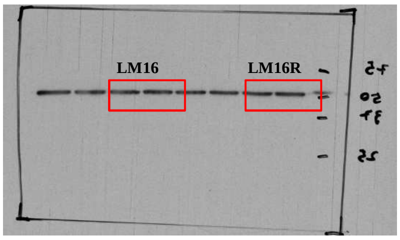

LM36

GPX4

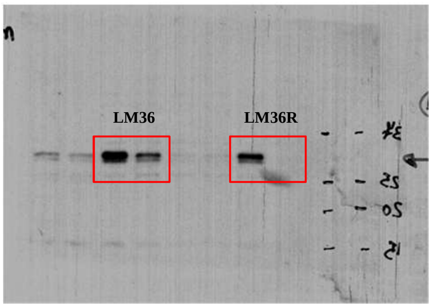

β-Tubulin

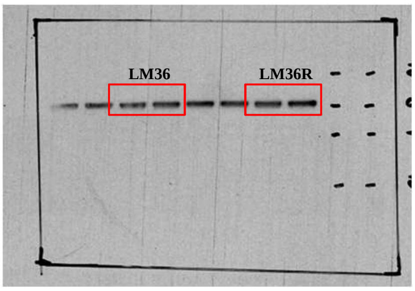

Western blot images for Figure 7

Condition	GPX4	Tubulin	Ratio
**LM16 Standard**	15437184	14267421	1,081988399
**LM16 lipid depleted**	15709280	17501559	0,89759318
**LM16R Standard**	20834860	15954759	1,305871182
**LM16R lipid depleted**	18472032	16582247	1,11396435
**Condition**	**GPX4**	**Tubulin**	**Ratio**
**LM36 Standard**	13483618	10364954	1,300885465
**LM36 lipid depleted**	4595338	11515650	0,399051552
**LM36R Standard**	8399756	8292588	1,012923348
**LM36R lipid depleted**	387182	11320376	0,034202221

Densitometric analysis was used to measure the band intensity. The relative expression levels (ratio between the band intensity of GPX4 and the corresponding band intensity of tubulin) was determined by ImageQuant 5.2 software.

Western blot images for Figure 7
