# Supplementary material for: Targeting of the Lipid Metabolism Impairs Resistance to BRAF Kinase Inhibitor in Melanoma
Source: Front Cell Dev Biol. 2022 Jul 13;10:927118. doi: 10.3389/fcell.2022.927118 (PMC9326082; doi:10.3389/fcell.2022.927118)
Supplement: Supplementary file 1 [file DataSheet1.ZIP › Vergani E_revised supplementary material 10-6-22/Vergani E-Supplemental Table S3.docx]

**Supplementary Table S3.** Percentage of each SFA, MUFA and PUFA in TG and CE.

|  |  | **LM16** | | **LM16R** | | **LM47** | | **LM47R** | | **LM36** | | **LM36R** | |
| --- | --- | --- | --- | --- | --- | --- | --- | --- | --- | --- | --- | --- | --- |
|  |  | **Mean** | **SD** | **Mean** | **SD** | **Mean** | **SD** | **Mean** | **SD** | **Mean** | **SD** | **Mean** | **SD** |
| **TG (%)** | **14.0** | 8.79 | 0.84 | 4.01 | 0.55 | 6.75 | 0.81 | 2.30 | 1.94 | 3.58 | 0.27 | 3.24 | 0.16 |
|  | **16.1** | 9.71 | 1.61 | 6.75 | 0.01 | 1.72 | 0.98 | 4.65 | 2.03 | 4.29 | 0.57 | 2.77 | 0.41 |
|  | **16.0** | 37.61 | 0.21 | 33.95 | 0.05 | 50.98 | 1.04 | 32.00 | 1.57 | 43.65 | 3.90 | 40.54 | 3.09 |
|  | **18.2** | 0.75 | 0.58 | 1.24 | 0.22 | 2.54 | 2.30 | 2.48 | 0.38 | 2.30 | 1.39 | 5.28 | 2.37 |
|  | **18.1** | 34.01 | 0.29 | 38.60 | 0.57 | 4.39 | 1.83 | 34.50 | 2.30 | 27.91 | 2.79 | 22.36 | 4.26 |
|  | **18.0** | 6.29 | 1.28 | 11.61 | 1.05 | 29.14 | 0.79 | 20.51 | 7.43 | 14.51 | 1.87 | 22.23 | 4.27 |
|  | **20.2** | 1.03 | 0.15 | 1.67 | 0.09 | 1.22 | 0.14 | 1.75 | 0.17 | 1.60 | 0.15 | 1.37 | 0.25 |
|  | **20.1** | 1.40 | 0.00 | 1.34 | 0.03 | 1.03 | 0.01 | 0.85 | 0.06 | 1.28 | 0.04 | 0.81 | 0.06 |
|  | **20.0** | 0.33 | 0.30 | 0.40 | 0.02 | 1.03 | 0.33 | 0.54 | 0.15 | 0.74 | 0.05 | 1.09 | 0.06 |
|  | **24.0** | 0.08 | 0.06 | 0.42 | 0.05 | 1.20 | 0.24 | 0.43 | 0.10 | 0.15 | 0.26 | 0.31 | 0.53 |
|  | **SFA** | 53.10 | 0.60 | 50.40 | 0.40 | 89.10 | 0.60 | 55.77 | 4.10 | 62.63 | 3.51 | 67.41 | 6.52 |
|  | **MUFA** | 45.12 | 1.30 | 46.68 | 0.50 | 7.14 | 2.80 | 40.00 | 4.30 | 33.48 | 2.73 | 25.94 | 4.02 |
|  | **PUFA** | 1.78 | 0.70 | 2.91 | 0.13 | 3.76 | 2.10 | 4.23 | 0.20 | 3.90 | 1.53 | 6.65 | 2.58 |
| **CE (%)** | **14.0** | 5.40 | 0.16 | 3.57 | 0.45 | 4.41 | 0.81 | 4.22 | 1.77 | 5.74 | 1.40 | 4.13 | 1.14 |
|  | **16.1** | 5.93 | 0.94 | 4.95 | 0.40 | 2.26 | 1.83 | 4.16 | 1.67 | 3.30 | 0.88 | 3.52 | 1.49 |
|  | **16.0** | 47.04 | 2.48 | 39.08 | 0.63 | 48.70 | 8.17 | 36.56 | 1.29 | 42.43 | 0.88 | 40.00 | 5.12 |
|  | **18.2** | 3.33 | 0.42 | 3.48 | 0.58 | 6.93 | 2.67 | 4.32 | 1.93 | 4.69 | 1.55 | 9.10 | 4.11 |
|  | **18.1** | 21.88 | 2.03 | 30.86 | 0.64 | 11.16 | 0.10 | 25.60 | 2.68 | 22.46 | 0.79 | 18.18 | 2.16 |
|  | **18.0** | 16.43 | 4.85 | 18.06 | 0.64 | 26.54 | 4.38 | 25.13 | 5.47 | 21.38 | 0.32 | 25.07 | 2.65 |
|  | **SFA** | 68.86 | 2.50 | 60.71 | 0.46 | 79.65 | 4.60 | 65.91 | 2.41 | 69.54 | 0.93 | 69.20 | 2.32 |
|  | **MUFA** | 27.81 | 2.90 | 35.81 | 1.04 | 13.42 | 1.93 | 19.77 | 4.34 | 25.76 | 1.43 | 21.70 | 1.98 |
|  | **PUFA** | 3.33 | 0.42 | 3.48 | 0.58 | 6.93 | 2.60 | 4.32 | 1.90 | 4.69 | 1.55 | 9.10 | 4.11 |

TG, triglycerides; CE, cholesteryl esters.
