# Supplementary material for: Targeting of the Lipid Metabolism Impairs Resistance to BRAF Kinase Inhibitor in Melanoma
Source: Front Cell Dev Biol. 2022 Jul 13;10:927118. doi: 10.3389/fcell.2022.927118 (PMC9326082; doi:10.3389/fcell.2022.927118)
Supplement: Supplementary file 1 [file DataSheet1.ZIP › Vergani E_revised supplementary material 10-6-22/Vergani E-Supplementary Table S4.docx]

**Supplementary Table S4.** Lipid starvation increases PLX4032 sensitivity of vemurafenib-resistant melanoma cells.^1^

| **PLX4032 (µM)** | **+ lipids** | | **- lipids** | |
| --- | --- | --- | --- | --- |
|  | **IC_50_ (µM)^2^** | **IC_30_ (µM)^3^** | **IC_50_ (µM)^2^** | **IC_30_ (µM)^3^** |
| **LM16R** | >10 | 9.45 | 9.6 | 6.4 |
| **LM36R** | 9.7 | 7.7 | 7.3 | 5.5 |
| **LM47R** | >10 | 9.3 | 8.9 | 6.3 |

^1^ Cell sensitivity was assessed by cell growth inhibition assay. Cells were seeded and 24 h later exposed to PLX4032 for 48h in presence or absence of lipids. Cells were then counted using a cell counter.

^2^ IC_50_ is the concentration of PLX4032 inhibiting 50% of cell growth.

^3^ IC_30_ is the concentration of PLX4032 inhibiting 30% of cell growth.
