# Supplementary figures and images for: Targeting of the Lipid Metabolism Impairs Resistance to BRAF Kinase Inhibitor in Melanoma
Source: Front Cell Dev Biol. 2022 Jul 13;10:927118. doi: 10.3389/fcell.2022.927118 (PMC9326082; doi:10.3389/fcell.2022.927118)

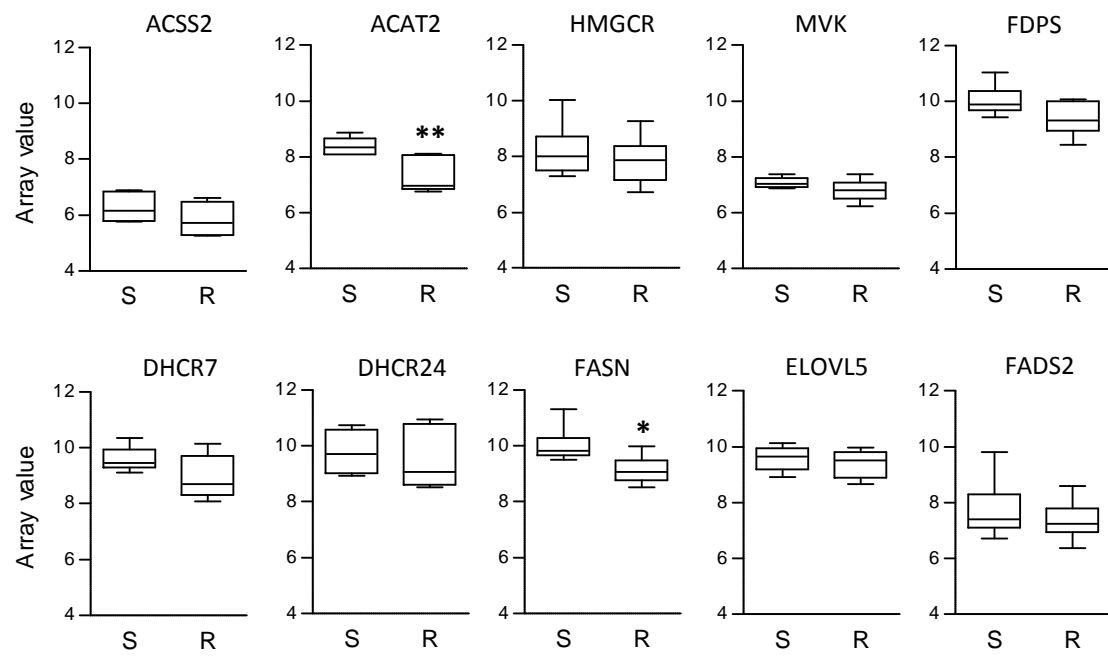

**Supplementary Figure S1**

Supplement: Supplementary file 1 [file DataSheet1.ZIP › Vergani E_revised supplementary material 10-6-22/Vergani E-Supplementary Figure S1.pdf]

**A**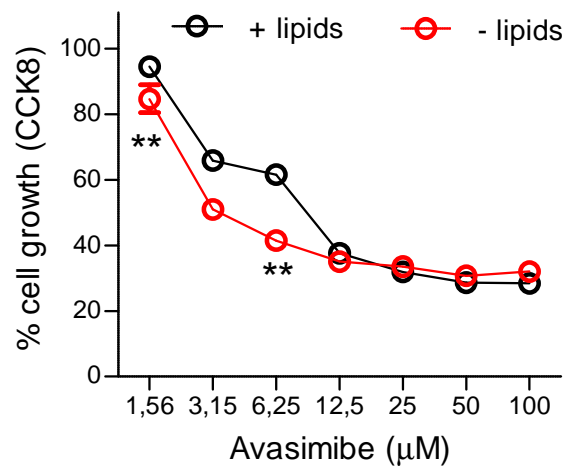**B**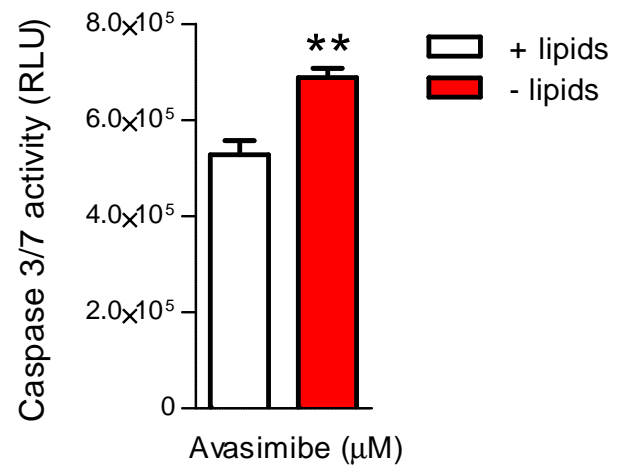

Supplementary Figure S2

Supplement: Supplementary file 1 [file DataSheet1.ZIP › Vergani E_revised supplementary material 10-6-22/Vergani E_Supplementary Figure S2.pdf]
